# Strong associations between fasting lipids and glucose concentrations and ALT levels strengthened with increasing ALT quantiles

**DOI:** 10.1186/s12944-024-02281-z

**Published:** 2024-09-12

**Authors:** Wei Liu, Lipu Shi, Mengmeng Yuan, Yonghui Zhang, Yalong Li, Chaofei Cheng, Junping Liu, Han Yue, Lemei An

**Affiliations:** 1 Department of Rheumatology and Clinical Immunology, Henan Provincial People’s Hospital, People’s Hospital of Zhengzhou University, Zhengzhou, Henan 450003 China; 2 Department of Gynecology, Henan Provincial People’s Hospital, Zhengzhou, Henan 450003 China; 3 Henan Key Laboratory of Stem Cell Clinical Application and Key Technology, Henan Provincial People’s Hospital, People’s Hospital of Zhengzhou University, Zhengzhou, Henan 450003 China; 4 Department of Infectious Diseases, Henan Provincial People’s Hospital, People’s Hospital of Zhengzhou University, Zhengzhou, Henan 450003 China

**Keywords:** Alanine transaminase (ALT), Aspartate transaminase (AST), Non-high-density lipoprotein cholesterol (non-HDL-C), HDL-C, Triglycerides, Glucose

## Abstract

**Background:**

A persistent redox state and excessive reactive species involved in carbohydrate and lipid metabolism lead to oxidative damage in the liver, however, how fasting plasma concentrations of lipids and glucose are associated with fasting blood levels of alanine transaminase (ALT) and aspartate transaminase (AST) remains to be evaluated in large-scale population.

**Methods:**

A cross-sectional study with 182,971 residents aged 18 to 92 years; multidimensional stratified analyses including quantile linear regression analysis and sex stratification were adopted to improve the quality of the evidence.

**Results:**

The associations between the concentrations of non-HDL-C and triglyceride and ALT levels were positive, stronger in males in each quantile of ALT levels and the coefficients expanded with increasing ALT levels at slopes of 3.610 and 5.678 in males and 2.977 and 5.165 in females, respectively. The associations between the HDL-C concentrations and ALT levels were negative, also stronger in males in each quantile and the coefficients expanded with increasing ALT levels at slopes of -7.839 in females and − 5.797 in males. The associations between glucose concentrations and ALT levels were positive, but stronger in females in each quantile and the coefficients expanded with increasing ALT levels at slopes of 1.736 in males and 2.177 in females, respectively. Similar pattern consist of relatively weaker coefficients and slops were observed between concentrations of non-HDL-C, triglyceride and glucose and AST levels. The associations between albumin concentration and concentrations of blood lipids and glucose were relatively steady across all quantiles.

**Conclusions:**

The dose dependent effect between blood concentrations of lipids and glucose and liver function changes suggests that excessive carbohydrate and lipid metabolism may cause subclinical liver damage. Long term sustained primary and secondary inflammatory factors produced in the liver might be transmitted to adjacent organs, such as the heart, kidneys, and lungs, to cause and/or exacerbate pathological changes in these visceral organs.

## Background

Carbohydrate metabolism (e.g., glycolysis, glycogen metabolism and gluconeogenesis), fatty acid metabolism (e.g., fatty acid synthesis, fatty acid oxidation and ketone body synthesis), cholesterol metabolism (e.g., digestion and recycling of cholesterol and fats), and bile acid metabolism occur mainly in the liver [1–3]. These rigorous and orderly physiological activities guarantee daily essential nutrition metabolism and an energy supply to our body [1–3]. However, these metabolic processes comprise a series of oxidation‒reduction reactions, which continuously produce reactive oxygen species (ROS) and reactive nitrogen species (RNS) [4, 5]. Although the human body has developed a complex antioxidant system to relieve oxidative stress, a persistent redox state and excessive reactive species may lead to oxidative damage to the liver itself [5]. In addition, hepatic glucose and lipid metabolism share common regulatory elements and metabolites and are closely related to inflammatory, proliferative and apoptotic signaling, which may also lead to low-grade inflammation in the liver [1–6].

The liver is the largest organ in the body, receives dual blood supplies from the hepatic artery and portal vein, and has two sets of output channels—the hepatic vein and biliary tract [7]. These anatomical characteristics ensure that the liver plays a central role in metabolic homeostasis. Thus, excessive ROS and RNS, as well as primary and secondary inflammatory factors produced in the liver during daily physiological and biochemical activities, can be easily transmitted to adjacent organs, such as the heart, kidneys, and lungs, to cause or exacerbate existing damage to these visceral organs [5]. Unfortunately, studies on whether overloaded nutrient metabolism may lead to hepatocyte damage and the consequences for adjacent organs are insufficient [5, 8]. Existing knowledge in this area is mostly derived from studies on nonalcoholic fatty liver disease (NAFLD) [9–11]. Alterations in the lipid storage and transport, insulin response, beta-oxidation, and autophagy and an imbalance in nuclear receptor and chemokines signaling are involved in hepatocyte damage, repair and regeneration [9–11]. Nevertheless, few studies have quantified the associations between changes in the concentrations of blood lipids and glucose and liver function [8]. This paucity is due to the following shortages in modern medicine. First, it is relatively easy to determine a disease with clinical manifestations (especially for diseases that are in a period of decompensation), but it is difficult to define the quantitative processes of a certain disease; that is, overloaded nutrient metabolism may cause subclinical liver damage, which cannot be anchored to a well-defined disease [12, 13]. Second, we often tend to infer pathogenesis in isolated and unilateral ways. For instance, trans-century studies have shown that disorders in lipid and glucose metabolism cause atherosclerotic cardiovascular disease (ASCVD) and diabetes mellitus [12, 13]. However, we have overlooked the possibility that liver damage caused by overloaded metabolism may cause low-grade systemic inflammation and hence also contribute to the pathogenesis of ASCVD. In addition, we have accepted “high-quality randomized controlled trials” as level A evidence, while cross-sectional studies have been considered to be level III or lower evidence [14]. As a result, the research community is increasingly suspicious of the conclusions of cross-sectional studies. Actually, high-quality cross-sectional studies were essential because it could provide important clues for randomized controlled trials and experimental studies [15].

The high and rising prevalence of metabolic diseases over the past two decades presents a large burden to us [16], which urgently requests us to expand our understanding of the pathophysiology that caused by overnutrition. To learn how fasting plasma concentrations of lipids and glucose are associated with subclinical liver damage, in this explorative cross-sectional study, we assembled 182,971 Chinese adults aged 18 to 92 years without known conditions that affect liver function or blood lipid and glucose metabolism, the association patterns between blood nutrition and liver function indexes were quantified by quantile regression analyses with multidimensional stratifications and strict quality control.

## Methods

### Ethical issues

This cross-sectional study followed the Strengthening the Reporting of Observational Studies in Epidemiology (STROBE) reporting guideline. The study was conducted following the standards of the Declaration of Helsinki and the study protocol was approved by the Review Board of Henan Provincial People’s Hospital, People’s Hospital of Zhengzhou University (HPPH2020-002). Informed consent was obtained from all subjects.

### Participants

**Fig. 1:**
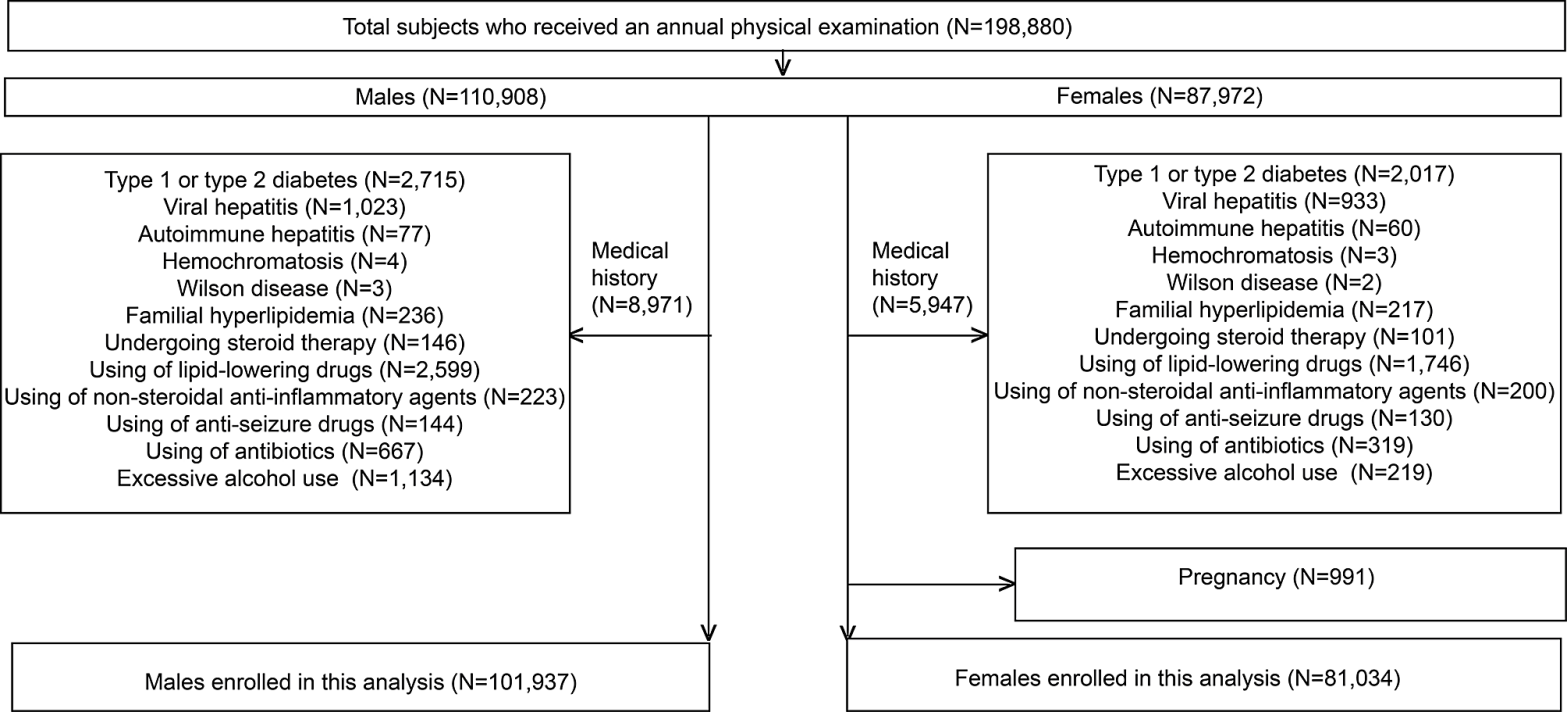
Flow chart of participants. In all, 198,880 residents were initially involved. Subjects with known conditions that affected liver tests as well as conditions that affected blood lipid and glucose metabolism were excluded. Ultimately, 182,971 subjects without known diseases were included in the present study

In all, 198,880 local residents (110,908 males and 87,972 females) who received an annual physical examination at our hospital between 2020 and 2023 were initially included in this study. Subjects who had known a parental history of diabetes (*n* = 4,732), type 1 diabetes or type 2 diabetes, viral hepatitis (*n* = 1,956), autoimmune hepatitis (*n* = 137), hemochromatosis (*n* = 7), Wilson disease (*n* = 5), a family history of hyperlipidemia (*n* = 453), and those with a history of treatment with lipid-lowering agents (*n* = 4345), steroid therapies (*n* = 247), nonsteroidal anti-inflammatory drugs (*n* = 423), antiseizure drugs (*n* = 274), and antibiotics (*n* = 986) were excluded; subjects with a history of excessive alcohol use (*n* = 1,353) and those who were pregnant (*n* = 991) at the time of the health checkup were also excluded from the study. Ultimately, 101,937 males and 81,034 females without known conditions that would affect liver tests and blood lipid and glucose metabolism were included in the analysis (Fig. 1).

### Clinical biochemical tests

All clinical biochemical parameters, including markers of hepatocellular injury, such as ALT and AST, and markers of liver function, such as albumin, and glucose and lipid profiles, were measured in venous blood samples obtained after overnight fasting in our hospital laboratory via daily routine procedures. All clinical biochemistry parameters were measured using Abbott Architect C16000 Clinical Chemistry Analyzer (Abbot, Maidenhead, Berkshire, UK). Albumin is measured by bromocresol purple-binding reaction. In ALT and AST measuring reactions, pyridoxal-5’-phosphate serves as a coenzyme in the amino transfer reaction, which. ensures full enzyme activation.

### Anthropometric measurements, questionnaires, and definitions

A body mass index (BMI) ≥ 30 kg/m2 indicated obesity. Smoking status and alcohol consumption were recorded using a questionnaire. A current smoker was defined as someone who smokes one or more cigarettes daily within 12 months. Excessive alcohol use was defined according the US CDC recommendation [17]. NAFLD was diagnosed according to the diagnosis and management of NAFLD [18].

### Study design and statistical analysis

This cross-sectional study has the following properties: (1) it has a large sample size of 182,971 subjects with a wide age range; (2) the series quality controls on the data analyses ensures the credibility of our result; and (3) multidimensional stratification analyses were performed to improve the level of evidence.

Compared with ordinary least squares regression, the results derived from the quantile regression analyses are interpreted in the context of the whole quantile of the distribution of the response variable rather than the mean, which provides a more comprehensive picture of the association between the liver tests and the levels of fasting glucose and lipid profiles [12, 19]. In addition, clinical biochemical parameters often fail to satisfy the assumption of normality and homoscedasticity of residuals [12]. So, we adopted the principles of quantile statistics in the analysis.

The association trends between each predictive variable and a response variable were explored by plotting the quantiles (0.1 to 0.9) of a response variable (vertical axis) against stratifications (equal 5% interval of measures) of predictive variables (horizontal axis) by sex. This preliminary plotting showed a rough linear correlation between the independent variable and the dependent variable in all quantile stratification.

In addition, blood lipids displayed strong correlations with each other. Thus, according to the biochemical principle and to avoid multicollinearity in the regressions, Non-HDL-C, HDL-C and triglyceride were selected to represent the lipid profile to evaluate their associations with the levels obtained by liver tests and were entered into the covariate groups solely during multivariable quantile regression modeling [12, 13]. In the male-adjusted models, age, current smoking status, NAFLD status, obesity status and non-HLD-C, HDL-C, or triglyceride levels were entered into the covariable group. In the female-adjusted models, age, NAFLD status, obesity status and non-HLD-C, HDL-C, or triglyceride levels were entered into the covariable group.

All statistical analyses were performed using IBM SPSS Statistics for Apple macOS (SPSS^®^ Statistics 26; IBM, NY), and the significance level was set at *P* < 0.05.

## Results

### Descriptions of the subjects

Table 1 describes the general characteristics of the participants by sex; the sex differences in biochemical test results are also provided. The prevalence of current smoking and NAFLD was greater in males. Conversely, the percentage of individuals with a BMI ≥ 30.0 was lower for males. The levels of ALT and AST and albumin concentration were greater in males; the concentrations of non-HDL-C, triglyceride and glucose were also greater in males, while the HDL-C concentration was lower in males.

**Table 1:** Statistical description of the general characteristics of the study participants

Variable	Males	Females	*P*
	*N* = 101,937	*N* = 81,034	
Age, years	33, 43, 52	31, 41, 50	*< 0.001*
BMI ≥ 30.0 kg/m², n (%)	14,577 (14.3)	12,074 (14.9)	*< 0.001*
Current smoker, n (%)	15,392 (15.1)	1,053 (1.3)	*< 0.001*
Nonalcoholic fatty liver disease, n (%)	17,023 (16.7)	9,157 (11.3)	*< 0.001*
Glucose, mmol/L	4.5, 4.9, 5.3	4.5, 4.8, 5.1	*< 0.001*
Total cholesterol, mmol/L	4.15, 4.72, 5.33	3.98, 4.51, 5.13	*< 0.001*
HDL-C, mmol/L	1.07, 1.23, 1.43	1.26, 1.46, 1.69	*< 0.001*
Non-HDL-C, mmol/L	2.89, 3.46, 4.07	2.49, 3.02, 3.64	*< 0.001*
Triglyceride, mmol/L	1.20, 1.72, 2.55	0.84, 1.18, 1.69	*< 0.001*
Total bilirubin, umol/L	10.4, 13.6, 17.5	8.7, 11.2, 14.3	*< 0.001*
Direct bilirubin, umol/L	2.6, 3.6, 4.9	2.2, 3.0, 4.2	*< 0.001*
Indirect bilirubin, umol/L	7.3, 9.9, 13.0	6.0, 8.1, 10.6	*< 0.001*
ALT, U/L	18, 25, 36	11, 15, 21	*< 0.001*
AST, U/L	18, 22, 27	16, 18, 22	*< 0.001*
Total protein, g/L	72.4, 75.0, 77.7	72.9, 75.5, 78.3	*< 0.001*
Albumin, g/L	46.8, 48.6, 50.4	45.7, 47.4, 49.2	*< 0.001*
Globulin, g/L	24.1, 26.3, 28.7	25.8, 28.1, 30.4	*< 0.001*

Continuous variates are presented as quartiles; categorical data are presented as numbers (percentages). *Abbreviations BMI* body mass index; *HDL-C* high-density lipoprotein cholesterol; *ALT* alanine transaminase; *AST* aspartate transaminase. Differences between groups were examined using a Mann‒Whitney U test and χ^2^ test according to the characteristics of data distribution

### Strong associations between blood concentrations of lipid and glucose and ALT levels become stronger with increasing ALT quantiles, regardless of sex

**Fig. 2:**
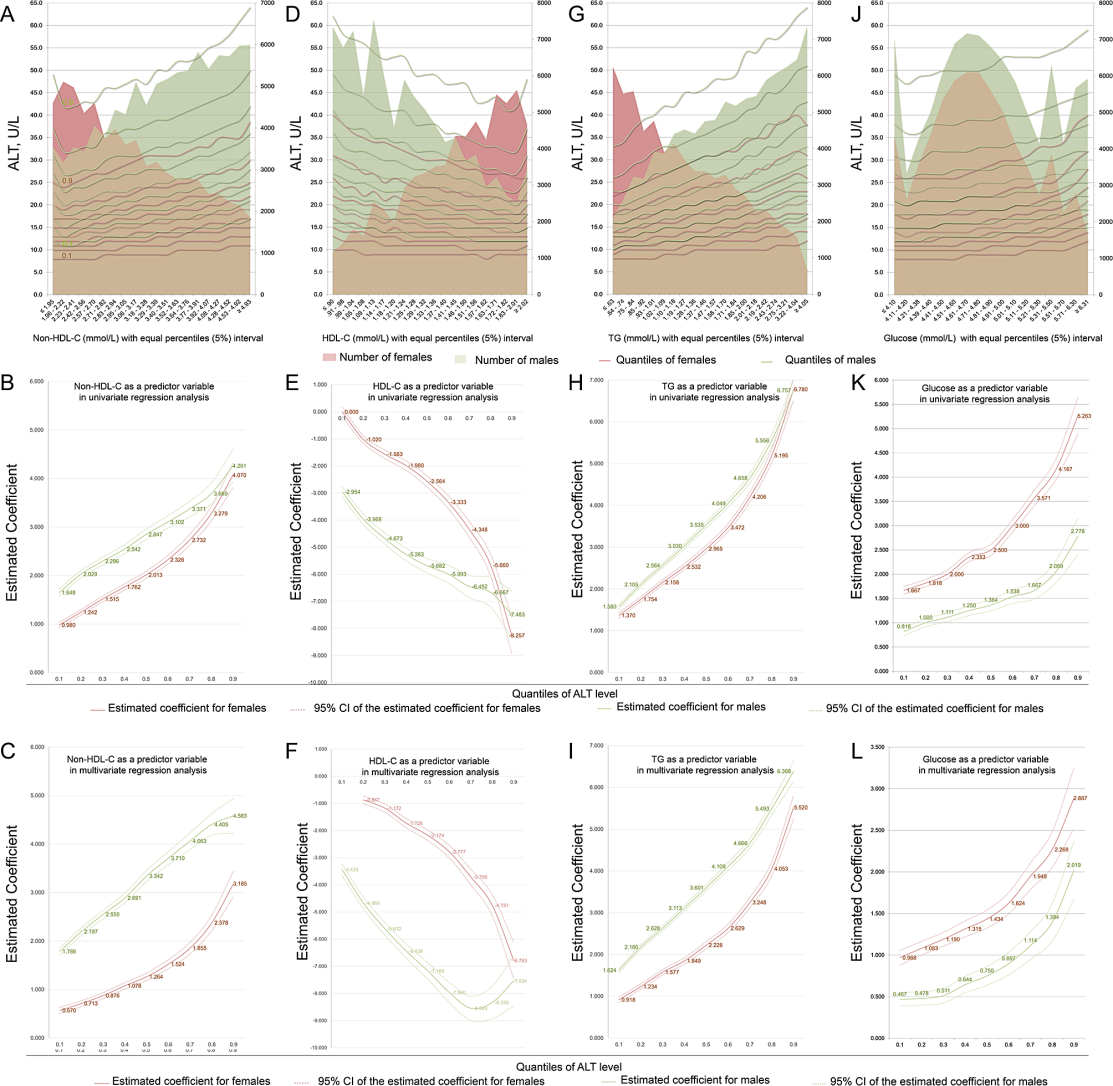
The associations between the concentrations of glucose and lipids and ALT levels. **A**, **D**, **G** and **J**, the association trends were first explored by plotting the quantiles of ALT levels (left vertical axis) against the glucose and lipid concentrations in 5% equal intervals (horizontal axis) by sex; shadows indicate the number of subjects in each 5% interval stratification (right vertical axis). **B**, **E**, **H** and **K**, UQLRA; the estimated coefficients (vertical axis) for the concentrations of non-HDL-C, HDL-C, triglycerides and glucose in association with ALT levels derived from quantile regressions are plotted against the quantiles of ALT levels (horizontal axis). **C**, **F**, **I** and **L**, multivariate linear quantile regression analysis. In the male-adjusted models, age, current smoking status, NAFLD status, obesity status and non-HLD-C, HDL-C, or triglyceride concentrations were entered into the covariable group; in the female-adjusted models, age, NAFLD status, obesity status and non-HLD-C, HDL-C, or triglyceride concentrations were entered into the covariable group

Preliminary plotting showed that strong associations between blood concentrations of lipid and glucose and ALT levels were strengthened with increasing quantiles of ALT level in both sexes (Fig. 2A, D, G and J).

Univariate quantile linear regression analysis (UQLRA) verified strong and positive associations between non-HDL-C concentrations and ALT levels, and these associations strengthened with increasing ALT quantiles in both sexes (Fig. 2B). Adjustments further enhanced the above associations in males and strengthened the coefficients from 1.786 (95% CI: 1.707 ~ 1.864, *P* < 0.001) in the 1st quantile to 4.583 (95% CI: 4.223 ~ 4.943, *P* < 0.001) in the 9th quantile. Adjustments attenuated the coefficients of females, but the coefficients still increased from 0.570 (95% CI: 0.507 ~ 0.633, *P* < 0.001) in the 1st quantile to 3.185 (95% CI: 2.920 ~ 3.450, *P* < 0.001) in the 9th quantile (Fig. 2B).

UQLRA also verified strong but negative associations between HDL-C concentrations and ALT levels, and these associations strengthened with increasing ALT quantiles in both sexes (except in the 1st quantile of females) (Fig. 2E). Adjustments attenuated the associations in females, and the coefficients displayed a negative increasing trend from − 0.867 (95% CI: -1.008~-0.725, *P* < 0.001) in the 2nd quantile to -6.783 (95% CI: -7.450~-6.116, *P* < 0.001) in the 9th quantile (Fig. 2F). Adjustments strengthened the associations in males, and the coefficients also showed a roughly negative decreasing trend from − 3.433 (95% CI: -3.642~-3.223, *P* < 0.001) in the 1st quantile to -7.534 (95% CI: -8.464~-6.605, *P* < 0.001) in the 9th quantile (Fig. 2F).

UQLRA showed very strong and positive associations between triglyceride concentrations and ALT levels, and the coefficients sharply increased as the ALT quantile increased in both males and females; in addition, the shapes of the estimated coefficient curves were similar between sexes (Fig. 2H). Adjustments further enhanced associations in males [coefficients ranging from 1.624 (95% CI: 1.568 ~ 1.679, *P* < 0.001) in the 1st quantile to 6.388 (95% CI: 6.147 ~ 6.630, *P* < 0.001) in the 9th quantile] but slightly attenuated coefficients in females [coefficients ranging from 0.918 (95% CI: 0.849 ~ 0.986, *P* < 0.001) in the 1st quantile to 5.520 (95% CI: 5.235 ~ 5.806, *P* < 0.001) in the 9th quantile] in all quantiles of ALT levels (Fig. 2I).

Additionally, UQLRA verified that glucose concentrations were positively associated with ALT levels in both sexes. These associations strengthened sharply in females whose ALT levels were above the 5th quantile and in males whose ALT levels were above the 7th quantile (Fig. 2K). Adjustments attenuated the associations both in males [coefficients ranging from 0.467 (95% CI: 0.386 ~ 0.547, *P* < 0.001) in the 1st quantile to 2.019 (95% CI: 1.668 ~ 2.371, *P* < 0.001) in the 9th quantile] and in females [coefficients ranging from 0.968 (95% CI: 0.881 ~ 1.056, *P* < 0.001) in the 1st quantile to 2.887 (95% CI: 2.525 ~ 3.250, *P* < 0.001) in the 9th quantile] and especially in the lower quantiles of ALT levels (Fig. 2L).

### Strong associations between concentrations of non-HDL-C, triglyceride and glucose and AST levels increase with increasing AST levels, regardless of sex

The preliminary plot shows that the association trends between the concentrations of non-HDL-C, triglyceride and glucose and AST levels were strong and were strengthened with increasing AST quantiles (Fig. 2A, G and J). Unusually, no unified trends were detected in the association between HDL-C concentrations and AST levels (Fig. 2D).

**Fig. 3:**
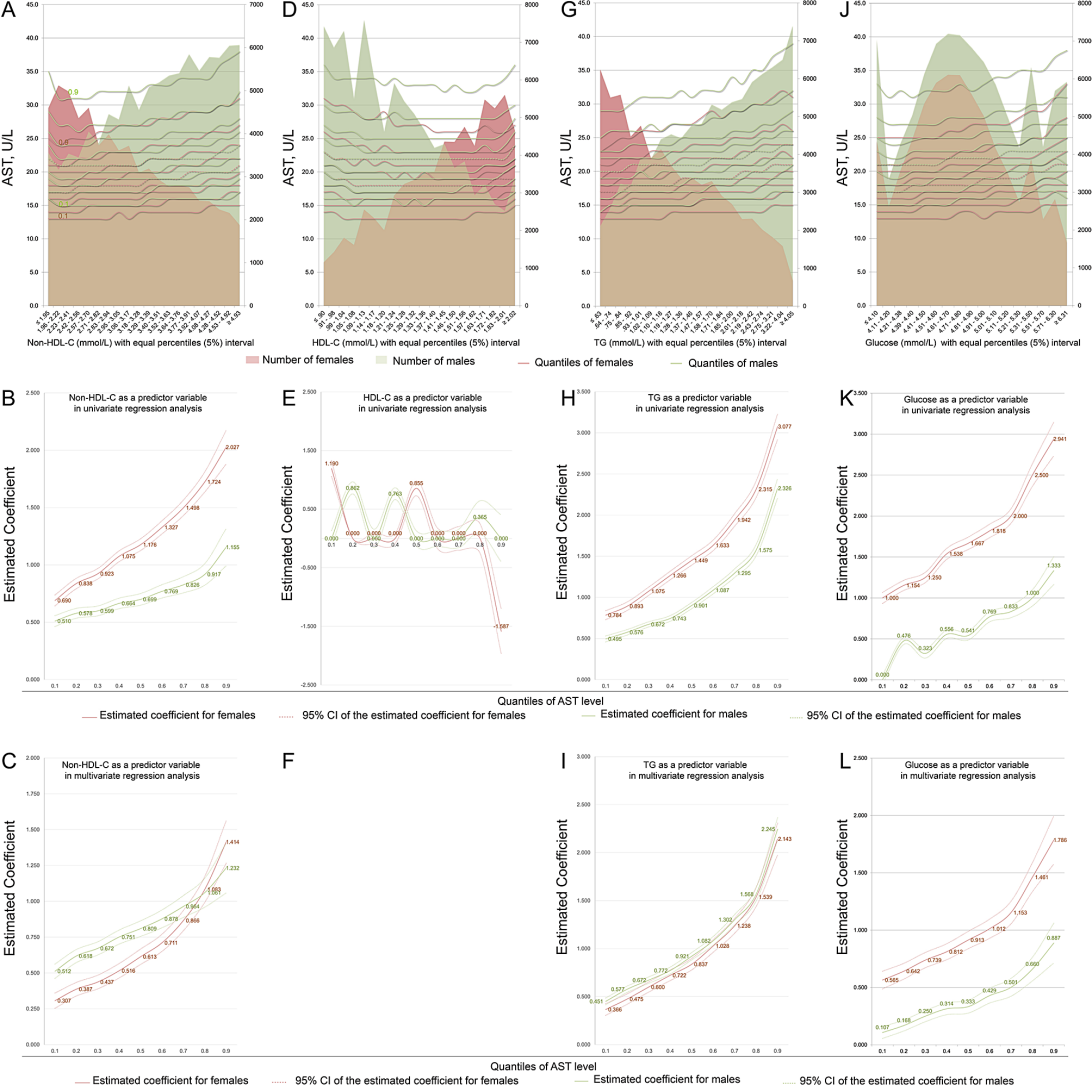
Associations between the concentrations of glucose and lipids and the AST levels. **A**, **D**, **G** and **J**, the association trends between AST levels and glucose and lipid concentrations were first explored by plotting the quantiles of AST levels (left vertical axis) against glucose and lipid concentrations in 5% equal intervals (horizontal axis) by sex; shadows indicate the number of subjects in each 5% interval stratification (right vertical axis). **B**, **E**, **H** and **K**, UQLRA; the estimated coefficients (vertical axis) for the concentrations of non-HDL-C, HDL-C, triglycerides and glucose in association with AST levels derived from quantile regressions are plotted against the quantiles of AST levels (horizontal axis). **C**, **F**, **I** and **L**, multivariate linear quantile regression analysis

UQLRA showed that non-HDL-C concentrations were positively associated with AST levels in both sexes; the coefficients increased with increasing quantiles of AST levels, and the associations were stronger in females than in males (Fig. 3B). Adjustments attenuated the coefficients in most quantiles of AST levels and narrowed the sex difference (Fig. 3C). The coefficients increased from 0.512 (95% CI: 0.462 ~ 0.562, *P* < 0.001) in the 1st quantile to 1.232 (95% CI: 1.058 ~ 1.406, *P* < 0.001) in the 9th quantile in males and from 0.307 (95% CI: 0.253 ~ 0.360, *P* < 0.001) in the 1st quantile to 1.414 (95% CI: 1.265 ~ 1.563, *P* < 0.001) in the 9th quantile in females.

UQLRA verified the unified associations between HDL-C concentrations and AST levels, and adjustments abolished all specious associations (Fig. 3E and F).

UQLRA showed strong and positive associations between triglyceride concentrations and AST levels in both sexes, and these associations strengthened with increasing AST levels (Fig. 3H). Adjustments attenuated the coefficients in all quantiles of AST levels in both sexes, and consequently, the coefficient curves of males and females were similar (Fig. 3I). The coefficients increased from 0.451 (95% CI: 0.415 ~ 0.486, *P* < 0.001) in the 1st quantile to 2.245 (95% CI: 2.126 ~ 2.364, *P* < 0.001) in the 9th quantile in males and from 0.366 (95% CI: 0.305 ~ 0.427, *P* < 0.001) in the 1st quantile to 2.143 (95% CI: 1.978 ~ 2.308, *P* < 0.001) in the 9th quantile in females.

Positive associations between glucose concentrations and AST levels were also observed in both sexes in the UQLRA, and the associations in females were stronger than those in males; after adjustment, the coefficient curves displayed more regular increasing trends with increasing AST level (Fig. 3K and L). The coefficients increased from 0.107 (95% CI: 0.055 ~ 0.159, *P* < 0.001) in the 1st quantile to 0.887 (95% CI: 0.714 ~ 1.061, *P* < 0.001) in the 9th quantile in males and from 0.565 (95% CI: 0.488 ~ 0.642, *P* < 0.001) in the 1st quantile to 1.786 (95% CI: 1.576 ~ 1.996, *P* < 0.001) in the 9th quantile in females.

### Blood concentrations of lipids and glucose are associated with albumin concentration

**Fig. 4:**
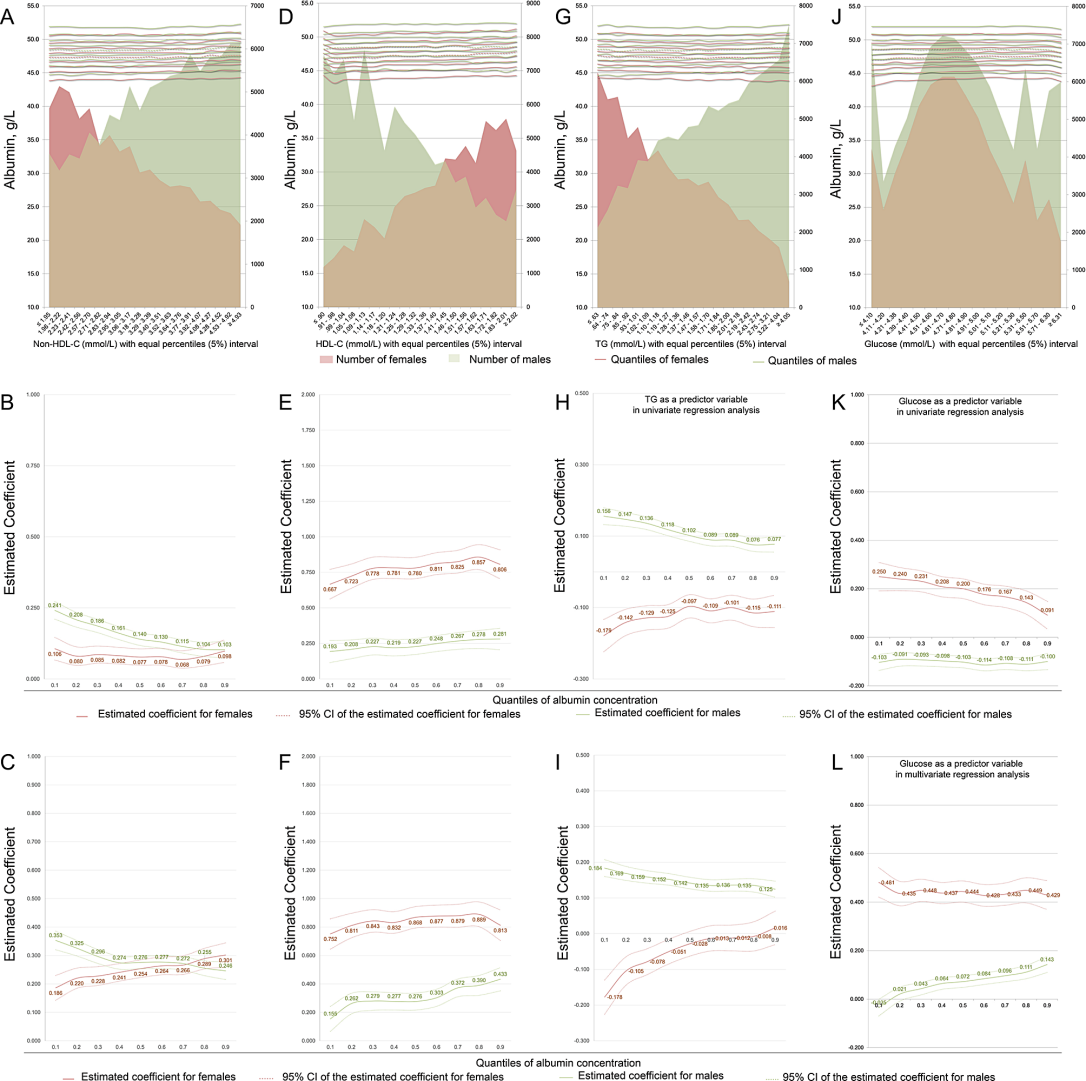
The associations between the concentrations of glucose and lipids and the serum albumin concentration. **A**, **D**, **G** and **J**, the association trends were first explored by plotting the quantiles of the albumin concentration (left vertical axis) against the concentrations of glucose and lipids in 5% equal intervals (horizontal axis) by sex; shadows indicate the number of subjects in each 5% interval stratification (right vertical axis). **B**, **E**, **H** and **K**, UQLRA; the estimated coefficients (vertical axis) for the concentrations of non-HDL-C, HDL-C, triglycerides and glucose in association with albumin concentrations derived from quantile regressions are plotted against the quantiles of the albumin concentration (horizontal axis). **C**, **F**, **I** and **L**, multivariate linear quantile regression analysis

Plotting the quantiles of albumin concentration against 5% equal interval levels of lipids and glucose by sex revealed relatively regular and gradual linear correlation trends across all quantiles (Fig. 4A, D, G and J).

UQLRA revealed that non-HDL-C concentrations were positively associated with the serum albumin concentration regardless of sex, with coefficients ranging between 0.103 and 0.241 for males and between 0.068 and 0.106 for females (Fig. 4B). Adjusted modeling strengthened the associations in both sexes, and the coefficients for males increased to 0.353 (95% CI: 0.320 ~ 0.386, *P* < 0.001) in the 1st quantile and 0.246 (95% CI: 0.215 ~ 0.277, *P* < 0.001) in the 9th quantile and display a gradual decrease trend; the coefficients for females increased to 0.186 (95% CI: 0.143 ~ 0.230, *P* < 0.001) in the 1st quantile and 0.301 (95% CI: 0.259 ~ 0.344, *P* < 0.001) in the 9th quantile and then gradually increased and display a gradual increase trend (Fig. 4C).

HDL-C concentrations were positively associated with albumin concentrations, as evaluated by the UQLRA, and the coefficients in all quantiles were approximately 3 times greater in women than in men (Fig. 4E). Adjustments enhanced the associations in both sexes in all quantiles of the serum albumin concentration except for the 1st quantile in males (Fig. 4F). The coefficients gradually increased from 0.752 (95% CI: 0.645 ~ 0.858, *P* < 0.001) in the 1st quantile to 0.813 (95% CI: 0.706 ~ 0.920, *P* < 0.001) in the 9th quantile in females and from 0.155 (95% CI: 0.067 ~ 0.242, *P* < 0.001) in the 1st quantile to 0.433 (95% CI: 0.351 ~ 0.515, *P* < 0.001) in the 9th quantile in males.

The UQLRA showed that blood triglyceride concentrations were positively associated with albumin concentrations in males, and the coefficients decreased slightly with increasing triglyceride quantiles (Fig. 4H). Adjustments had little effect on the association patterns (Fig. 4I). Triglyceride concentrations were negatively associated with albumin concentrations in females in the UQLRA (Fig. 4H); however, the negative associations remained significant only in quantiles 0.1 to 0.4 after adjustment (Fig. 4I).

Blood glucose concentrations were positively associated with albumin concentration in females and negatively associated with albumin concentration in males according to the UQLRA (Fig. 4K). Adjustments enhanced the associations in females, and the coefficients remained between 0.428 and 0.481; however, the associations became positive but weak in males through a quantiles of 0.2 to 0.9 of albumin concentrations (Fig. 4L).

### The ranking of the associations

To further quantify the association intensity as the quantile increased, linear equations were adopted to fit each of the 9 coefficients derived from all 24 quantile regressions. Table 2 summarizes stepping up in the quantile of the serum albumin concentration and levels of ALT and AST, the coefficients corresponding to each multivariable quantile linear regression could be increased to slope* quantile. With every 0.1 increase in the quantile of the ALT level, the coefficients associated with the plasma concentrations of non-HDL-C, HDL-C, triglycerides and glucose increased by 0.361, -0.580, 0.568 and 0.174 times, respectively, in males and by 0.298, -0.784, 0.517 and 0.218 times, respectively, in females, which suggests that the associations between the concentrations of non-HDL-C, HDL-C, triglyceride or glucose and ALT levels are strongest. The associations between the AST levels and albumin concentrations and the plasma indices were the 2nd and 3rd strongest, respectively.

**Table 2:** The slopes (coefficients) of the linear equations fitted the trends composed of the coefficients derived from multivariate quantile regression modeling

	Males				Females			
	Slope	Lower 95% CI	Upper 95% CI	*P*	Slope	Lower 95% CI	Upper 95% CI	*P*
Non-HDL-C								
Albumin	-0.114	-0.157	-0.071	*< 0.001*	0.128	0.106	0.149	*< 0.001*
ALT	3.610	3.387	3.833	*< 0.001*	2.977	2.139	3.815	*< 0.001*
AST	0.820	0.707	0.932	*< 0.001*	1.262	0.991	1.612	*< 0.001*
HDL-C								
Albumin	0.285	0.194	0.376	*< 0.001*	0.099	-0.014	0.212	*0.077*
ALT	-5.797	-8.222	-3.372	*< 0.001*	-7.839*	-10.182	-5.496	*< 0.001*
AST	#				#			
Triglyceride								
Albumin	-0.067	-0.085	-0.049	*< 0.001*	0.407*	0.068	0.746	*0.035*
ALT	5.678	5.052	6.304	*< 0.001*	5.165	3.752	6.577	*< 0.001*
AST	1.953	1.333	2.574	*< 0.001*	1.980	1.412	2.549	*< 0.001*
Glucose								
Albumin	0.150*	0.116	0.183	*< 0.001*	-0.035	-0.078	0.485	*0.096*
ALT	1.736	1.068	2.404	*< 0.001*	2.177	1.528	2.825	*< 0.001*
AST	0.869	0.647	1.090	*< 0.001*	1.395	1.006	1.785	*< 0.001*

*Notes* *the linear equation fits of coefficients in the quantiles with P<0.01 in the multivariate regression analyses; #no linear equation was fitted. For every one unit increase in x (each quantile of the concentrations of albumin and levels of ALT and AST) the predicted value of y (coefficients corresponding to the multivariate regression in each quantile) increases by the value of the slope

## Discussion

Given that the units of predictor and response variables are all mmol/L, our data show that 1 mmol/L changes within their ranges of non-HDL-C, triglyceride or glucose are associated with particular quantitative changes in each quantile of the ALT and AST levels by sex. In addition, our data also showed that these associations further strengthened as the quantiles of the response variables increased, especially for ALT and AST. Since a persistent redox state and excessive reactive species lead to oxidative damage to the liver itself [5], our quantile regression analyses defined a dose-response relationship between metabolic load and activity of liver enzymes in two aspects. Furthermore, although the concentrations of non-HDL-C, triglyceride and glucose were higher in males than in females, the above association patterns were similar between males and females, which suggests a common mechanism shared between males and females and supports the above dose-response relationship from a third perspective.

The associations between concentrations of non-HDL-C, HDL-C, triglyceride and glucose and ALT levels were similar to what observed in ASCVD studies, that is, higher concentrations of non-HDL-C, triglyceride and glucose and lower HDL-C concentration were more inclined towards onset of ASCVD [20]. This phenomenon suggests that metabolic related ASCVD pathogenesis may also occur in the liver. Considering the anatomical, structural, and functional characteristics of the liver, these primary and secondary liver damage products might be transmitted to adjacent organs and create a vicious cycle. For HDL-C, although no association was observed in multivariate quantile linear regression analyses when using AST as a response variate, the associations between HDL-C concentrations and ALT levels were negative, stronger in males in each quantile and strengthened with increasing ALT levels at slopes of -7.839 in females and − 5.797 in males. This result suggests that the metabolic damage in liver caused by high concentrations of non-HDL-C, triglyceride and/or glucose might reduce HDL-C output. Because HDL-C concentration is lower in males (Table 1), this possibility is more likely to occur in the male population. The glucose concentration is higher in male population, however, the association between glucose concentration is stronger in females in each quantile of the levels of ALT and AST and albumin concentration, suggesting that metabolism of glucose is more closely associated with liver function changes in females with unknown reason. Taken together, synthetic overload, recycling and degradation of lipids and glucose metabolism might cause sex-specific metabolic pathophysiology in the liver.

Although ALT and AST are not hepatocyte-specific enzymes, they are the most commonly used markers of hepatocyte injury because acute hepatitis, toxic injury or ischemic injury in the liver results in the leakage of these enzymes into the circulation [21, 22]. Normal values for laboratory tests of ALT and AST are defined as those found in 95% of a population [23]. Thus, the clinical use of these two parameters is mainly limited to acute liver injuries [21–23]. Our data suggest the possibility that ALT and AST levels within normal reference intervals might also be served as subclinical liver damage markers. Interestingly, although our results showed that the concentrations of HDL-C were negatively associated with the ALT levels in males and females (except in the 1st quantile of the ALT level) and that the associations strengthened with increasing ALT level, no association was observed between HDL-C and AST after adjustment. This might be because periportal hepatocytes have relatively more ALT, hepatocytes near the central vein have more AST, and functional HDL-C might be abundant in the area near the portal vein [23].

Our results are consistent with most basic studies and suggest that excessive carbohydrate and lipid metabolism leads to liver damage [5, 24, 25]. So far, very few studies have focused on a similar theme [8, 26], two existing reports showed that (1) both high LDL and HDL were associated with significantly higher odds of elevated liver enzymes in the general US adult population [8]; (2) triglyceride concentrations were positively associated with elevated ALT levels [26]. Although these conclusions may seem similar to ours, our report differed to these two reports from study design to sample size, the two reports had only adopted categorical variable to perform multivariable analysis which may lost a lot of important information regarding the associations. Our report has following merits: (1) large-scale sample size with a wide age range; (2) the series quality controls on the data analyses; and (3) multidimensional stratification. These strategies ensured the credibility of our result and improved the level of evidence.

Albumin is synthesized in the liver, and thus the plasma albumin concentration can serve as an index of liver synthetic capacity [27, 28]. Nevertheless, the albumin concentration in the plasma is difficult to interpret due to the high synthetic capacity of the liver, its half-life of three weeks in the plasma and because two-thirds of the body’s albumin is located in the extravascular and extracellular space [29, 30]. In this report, the quartiles of the serum albumin concentration were 46.8, 48.6 and 50.4 g/L in males and 45.7, 47.4 and 49.2 g/L in females, which suggests that our participants were adequately nourished. In this situation, positive associations between concentrations of non-HDL-C, HDL-C, triglyceride and glucose and albumin concentrations indicate positive feedback between abundant nutrition and enhanced synthetic ability.

The shortcomings of this study are as follows: (1) the data involve a wide range of medical science, and thus we are unable to explain all the results in a detailed and reasonable manner; and (2) this explorative study only revealed that blood lipids and glucose levels are associated with hepatocyte damage but we have not evaluated the possible effect to adjacent organs.

## Conclusion

Higher concentrations of non-HDL-C, triglyceride and glucose are associated with higher levels of ALT and/or AST in general population. Long-term and sustained excessive carbohydrate and lipid metabolism may cause subclinical liver damage.

## Data Availability

All data could be requested from corresponding author. Qualified researchers should submit a proposal to the corresponding author outlining the reasons for requiring the data. Use of data must also comply with the requirements of our institutes.

## Abbreviations

95%CI: 95% confidence intervals
ALT: Alanine transaminase
ASCVD: Atherosclerotic cardiovascular disease
AST: Aspartate transaminase
BMI: Body mass index
NAFLD: Nonalcoholic fatty liver disease
non-HDL-C: Non-high-density lipoprotein cholesterol
ROS: Reactive oxygen species
RNS: Reactive nitrogen species
STROBE: Strengthening the Reporting of Observational Studies in Epidemiology
UQLRA: Univariate quantile linear regression analysis
US CDC: Center for disease control and prevention of the united states
